# Age and African-American race impact the validity and reliability of the asthma control test in persistent asthmatics

**DOI:** 10.1186/s12931-018-0858-0

**Published:** 2018-08-15

**Authors:** Allison J. Burbank, Krista Todoric, Pamela Steele, Jonathan Rosen, Haibo Zhou, Marcia Frye, Ceila E. Loughlin, Sally Ivins, Katherine Mills, Lauren Dembnicki Massey, Bryce B. Reeve, Michelle L. Hernandez

**Affiliations:** 1 Division of Pediatric Allergy, Immunology and Rheumatology, University of North Carolina, Chapel Hill, NC USA; 2 Penn State Hershey Allergy, Asthma & Immunology, Hershey, PA USA; 3 Department of Biostatistics, Gillings School of Global Public Health, University of North Carolina, Chapel Hill, NC USA; 4 Naval Medical Center Portsmouth, Portsmouth, VA USA; 5 Division of Pediatric Pulmonology, University of North Carolina, Chapel Hill, NC USA; 6 Center for Environmental Medicine, Asthma and Lung Biology, University of North Carolina, 104 Mason Farm Road, Chapel Hill, NC 27599-7310 USA; 7 Sandhills Pediatrics, Southern Pines, NC USA; 8 Department of Population Health Sciences, Duke University School of Medicine, Durham, NC USA

**Keywords:** Asthma, The asthma control test (ACT), African-American, Adolescent, Questionnaire, Control, Validity, Reliability

## Abstract

**Background:**

The Asthma Control Test (ACT) is widely used to assess asthma control, yet the validity and reliability of the test have not been specifically evaluated in adolescents or African-Americans. We conducted a prospective psychometric study of the ACT in African-American (AA) and non-African-American (nAA) adolescents with persistent asthma, with emphasis on the clinical utility of the test for medical decision making.

**Methods:**

Participants completed the ACT and performed spirometry. A physician conducted a guidelines-based assessment of asthma control, blinded to the ACT score. Study procedures were repeated 6–8 weeks later. The ACT-based asthma control assessment was compared to physician assessment.

**Results:**

For baseline and follow-up visits, internal consistency, as measured using Cronbach’s alpha, was 0.80 and 0.81 in AA teens and 0.80 and 0.83 in nAA teens. Intraclass correlation coefficients were 0.59 and 0.76 in AA and nAA teens, respectively, with stable asthma control over time. Agreement between ACT and physician assessment was moderate in AA teens and fair in nAA teens. An ACT score of ≤19 showed reduced sensitivity for not well controlled asthma in both groups, while a score of ≤21 had the greatest area under the ROC curve. ACT scores were marginally responsive to change in control status.

**Conclusions:**

Concerns for the ACT’s ability to detect uncontrolled asthma in adolescents emphasizes the need for a more comprehensive evaluation of asthma control in clinical settings. A higher threshold ACT score to define not well controlled asthma may be needed if the ACT is to be used for medical decision making.

**Trial registration:**

ClinicalTrials.gov: NCT02671643, NCT02662413.

**Electronic supplementary material:**

The online version of this article (10.1186/s12931-018-0858-0) contains supplementary material, which is available to authorized users.

## Background

Asthma is a common chronic illness with significant morbidity and mortality, despite the availability of evidence-based treatment guidelines. There continues to be noticeable disparity in asthma outcomes among African-Americans, with rates of asthma-related healthcare utilization and death approximately 2 to 3 times the rates seen in Caucasians [1]. Asthma among African-Americans is disproportionally not well controlled [2–4]. Adolescence may add additional risk, as children are becoming more independent from their parents and engaging in risk-taking behaviors [5]. Teenagers with uncontrolled asthma are more likely to “normalize” their asthma and have a higher threshold for reporting symptoms and initiating treatment [6].

The National Asthma Education and Prevention Program (NAEPP) guidelines recommend assessment of asthma control at each asthma visit, including frequency of asthma symptoms and rescue medication use, activity limitation, unscheduled healthcare visits, and spirometry measurement [7], and have incorporated use of standardized asthma questionnaires including the Asthma Control Test (ACT)™ into this assessment. Asthma questionnaires have taken a leading role in clinical management and are also frequently used in research for subject selection and for measurement of treatment effects. The self-administered questionnaire assessing impairment during the previous four weeks was validated for use in those 12 years and older [8], but concerns have been raised about the performance of the ACT in adolescents and ethnic minority populations [9], who were under-represented in previous validation studies. While a score of 19 or less out of a possible 25 points showed the greatest sensitivity and specificity for uncontrolled asthma [8], these findings were derived from predominantly Caucasian adult study populations, with median ages of 35 to 45 years [8, 10]. Studies examining the use of the ACT in adolescents of European and Mexican ancestry have reported higher optimal cut points to distinguish well controlled from uncontrolled asthma [11–13]. This divergence may be in part due to cultural and developmental or age-related differences in the way symptoms are perceived and reported, as well as differences in health literacy when compared to Caucasian adults. While questionnaires like the ACT were initially developed to serve as *one component* of the asthma control assessment, many busy practices have embraced the ACT as essentially a replacement for the recommended multidimensional assessment. This is especially concerning given the questions surrounding the performance of the ACT in teens and minorities who may already be more likely to have their asthma severity underestimated by healthcare providers [14–17]. To avoid inappropriate medical management, it is imperative that tools like the ACT, which rely solely on patient-reported data, be validated in the populations in which they are frequently used [18].

To address these questions, we conducted a prospective study to evaluate the validity and reliability of the ACT in African-American and non-African-American adolescents with persistent asthma, with emphasis on the clinical utility of the ACT for asthma management.

## Methods

### Subjects/recruitment

Participants were recruited from the University of North Carolina’s pediatric allergy and pulmonology subspecialty clinics and general pediatric clinics. We enrolled adolescents ages 12 to 18 years with a physician diagnosis of persistent asthma who were using a controller medication (inhaled corticosteroid, combination inhaled corticosteroid/long acting beta agonist, or leukotriene receptor antagonist). Children were included in the African-American (AA) or non-African-American (nAA) cohorts based on their self-identification of racial/ethnic background. Children were excluded from study if they had a diagnosis of pulmonary disease other than asthma (such as vocal cord dysfunction or cystic fibrosis), if they were unable to perform spirometry, or if they were unable to speak and read English. Written informed consent and assent were obtained from participating children and their guardians (if the participant was less than 18 years of age).

### Study protocol

Each participant presented for two study visits. At the baseline visit, teens completed the ACT questionnaire without the help of the parent/guardian. The coordinator obtained past medical history, reviewed home medications, allergies, review of systems, and performed spirometry. Atopy was defined by a documented history of sensitization to 1 or more aeroallergens (by skin prick testing or serum IgE testing), history of allergic rhinitis, food allergy, or atopic dermatitis. A study physician, blinded to the ACT score, then obtained a standardized asthma history assessing the frequency of asthma symptoms and rescue medication use, activity limitation, and asthma-related healthcare utilization over the prior 4 weeks (see Additional file 1: Table S1 in the Online Repository) in addition to a focused physical exam. Taking into account history, patient-reported symptoms (other than the ACT), physical exam findings, and spirometry measurements, the study physician made an assessment of asthma control as being either well controlled or not well controlled. Participants returned six to eight weeks later for their follow up study visit, and the same procedures were repeated. The study protocol was approved by the University of North Carolina’s institutional review board.

### ACT measurements

#### Reliability

To estimate internal consistency reliability, or the consistency among responses to items in the ACT at a single assessment point, Cronbach’s coefficient alpha was computed for baseline and follow-up visits for AA and nAA teens. A value of 0.70 or greater is considered an acceptable reliability estimate for group-level assessment of asthma control (such as in a clinical trial), and a threshold of 0.90 or greater is recommended for individual-level assessments as would be made for routine clinical care and screening [19–21]. Intraclass correlation coefficients (ICC) were calculated to estimate test-retest reliability, or the degree to which ACT scores are similar over time when there is no change in the participants’ asthma control. An ICC of less than 0.5 is associated with poor reliability, 0.5–0.75 is fair, 0.75–0.9 is good, and greater than 0.9 is associated with excellent reliability [22].

#### Criterion and construct validity

We evaluated criterion validity by computing Cohen kappa (κ) statistics, which measure the agreement between asthma control assessments (ACT vs physician assessment) [23, 24]. κvalues ≤0 indicate no agreement, 0.01–0.2 slight agreement, 0.21–0.4 fair agreement, 0.41–0.6 moderate agreement, 0.61–0.8 substantial agreement, and 0.81–1.0 almost perfect agreement. Pearson correlation coefficients were calculated between baseline ACT scores and spirometry measurements. Construct validity (specifically, known groups validity) was measured by comparing 1) mean ACT scores of teens with well controlled asthma to those with not well controlled asthma using Student’s t test, and 2) mean ACT scores between groups differing by severity of airway obstruction (measured by spirometry) by ANOVA.

#### Screening accuracy

We determined the accuracy of the ACT for detecting not well controlled asthma in AA and nAA teens using receiver operating characteristic (ROC) analyses. Physician assessment of asthma was used as the “gold standard”. Sensitivity, specificity, positive and negative predictive values, and areas under the ROC curve were calculated for cut point scores from ≤10 to ≤24.

#### Responsiveness

Responsiveness of the ACT to change in asthma control status was determined by calculating Pearson correlation coefficients for the relationship between change in ACT score and 1) change in physician assessment of asthma control and 2) change in spirometry measurements. Mean change in ACT scores was compared between groups whose physician-assessed asthma control improved, remained stable, or worsened using ANOVA.

## Results

### Participants

Fifty-four AA teens and 36 nAA teens with persistent asthma participated in the study. Fifty-two AA teens and 35 nAA teens completed both study visits. One AA teen and 3 nAA teens were receiving step 1 therapy despite a diagnosis of persistent asthma and were excluded from the analysis. The AA and nAA groups were similar in terms of age, sex distribution, BMI, % predicted FEV_1_, and presence of atopy (Table 1). Asthma severity for the two groups was approximated by treatment step, with 85% of the AA group and 76% of the nAA group requiring Step 3 therapy or higher, indicating at least moderate persistent asthma in the majority of participants [7]. Eight AA and 5 nAA participants were receiving anti-IgE monoclonal antibody therapy during the study period. At the baseline visit, 60% of AA teens and 42% of nAA teens had asthma that was not well controlled.

**Table 1:** Subject Demographics

	AA (*N* = 53)	nAA (*N* = 33)
Age in years, median (range)	13.8 (12–18)	13.5 (12–18)
Sex – Feale, n(%)	26 (50%)	14 (42%)
Race/Ethnicity to which subject self-identifies	53 African-American	25 Caucasian 2 Asian 5 Hispanic/Latino 1 Native American
BMI %tile for age/sex, median (range)	72 (3–99)	72 (1–99)
% predicted FEV_1_, median (range)	93 (50–138)	92 (54–114)
% predicted FEV_1_, n (%)		
< 60%	1 (2)	1 (3)
60–79%	8 (15)	2 (6)
80–100%	26 (49)	23 (70)
> 100%	18 (34)	7 (21)
NAEPP Treatment Step, n (%)	Step 2–6 (11)	Step 2–7 (21)
	Step 3–11 (21)	Step 3–10 (30)
	Step 4–8 (15)	Step 4–7 (21)
	Step 5–26 (49)	Step 5–7 (21)
	Step 6–0 (0)	Step 6–1 (3)
	Unknown – 2 (4)	Unknown – 1 (3)
Asthma Control, n (%)	Well controlled – 21 (40)	Well controlled – 19 (58)
	Not well controlled – 32 (60)	Not well controlled - 14 (42)
History of atopic disease, N (%)	49 (92)	30 (91)

### Reliability

Cronbach’s coefficient alpha was computed for both the AA and nAA groups at baseline and follow-up visits. Cronbach’s alpha of the ACT was 0.80 and 0.81 for the AA teens at baseline and follow-up visits, respectively. For the nAA teens, Cronbach’s alpha was calculated at 0.80 and 0.83 for baseline and follow up visits, respectively. Among those with stable asthma control during the study, ICC was 0.59 in the AA group (37 observations) and 0.76 in the nAA group (19 observations).

### Criterion and construct validity

The previously established cut point of ≤19 was associated with a κof 0.43 in the AA group and 0.36 in the nAA group, consistent with moderate and fair agreement between physician and ACT assessment of asthma control, respectively [23, 24]. These findings are comparable to a recent systematic review and meta-analysis of studies examining the performance of the ACT [25]. To test construct validity, we compared the mean ACT scores for those with well controlled asthma to those with not well controlled asthma and found that in both AA and nAA teens, those with not well controlled asthma had statistically significantly lower ACT scores than those with well controlled asthma (Table 2). For the AA group at baseline, mean ACT score difference was 4.13 (well controlled – not well controlled) (*p* < 0.0001). Similarly for the nAA group, the mean ACT score difference was 4.58 points (*p* = 0.0002). Baseline visit ACT scores were poorly correlated with baseline spirometry measurements in both AA and nAA teens (see Additional file 1: Table S2 in the Online Repository).

**Table 2:** Discriminant validity of the ACT at the first study visit in AA and nAA adolescents

	African-Americans				Non-African-Americans			
Physician Assessment of Asthma								
	n	Mean (SD) ACT Score		p	n	Mean (SD) ACT Score		p
Controlled	21	22.4 (2.2)		< 0.0001	19	22.8 (2.4)		0.0003
Not well controlled	32	18.3 (3.7)			14	18.2 (4.1)		
Spirometry								
	n	Mean (SD) ACT Score	F	p	n	Mean (SD) ACT Score	F	p
% predicted FEV_1_								
< 60%	1	24 (0)	0.5	0.68	1	13 (0)	1.51	0.23
60–79%	8	19.25 (2.32)			2	21.5 (0.71)		
80–100%	26	19.73 (3.92)			23	21.17 (4.05)		
> 100%	18	20.17 (4.20)			7	20.43 (3.16)		
FEV_1_/FVC								
< 60%	0	–	–	0.32^†^	1	13 (0)	2.83	0.07
60–79%	25	19.72 (3.04)			11	20.09 (4.53)		
80–100%	28	20.04 (4.39)			21	21.52 (3.20)		
> 100%	0	–			0	–		
FEF_25–75_								
< 60%	21	19.62 (3.12)	0.88	0.46	5	18.2 (3.96)	0.89	0.46
60–79%	12	21.33 (2.64)			12	21 (4.41)		
80–100%	9	18.78 (5.17)			10	21.3 (3.37)		
> 100%	11	19.73 (4.69)			6	21.67 (3.67)		

^†^Mann-Whitney U test

### Screening accuracy

**Fig. 1:**
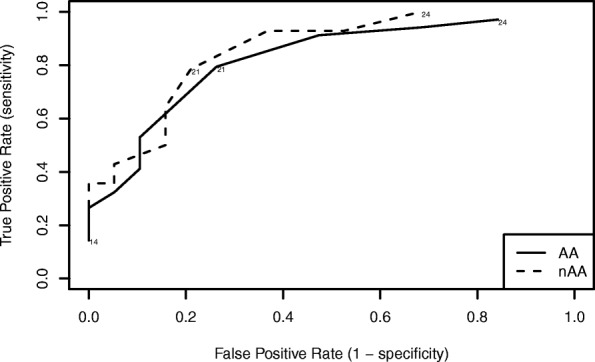
Receiver operating characteristic (ROC) curves generated from ACT scores from the first study visit for AA and nAA teens indicate a score of ≤21 achieved the optimal balance of sensitivity and specificity for detection of not well controlled asthma and the largest area under the ROC curve

In AA teens, the currently recommended cut point score of ≤19 to indicate not well controlled asthma showed reduced sensitivity (56%) and NPV (58%) compared to previous studies (Table 3) [8, 10]. A score of ≤21 achieved the greatest area under the ROC curve at 0.76 with sensitivity and PPV of 81% and specificity and NPV of 71% (Fig. 1). Increasing the cut point to ≤21 improved the level of agreement between ACT and physician assessment, or κ, from 0.43 to 0.53. Similar to AA teens, for nAA teens a cut point score of ≤21 provided the maximum area under the ROC curve at 0.79 with sensitivity and specificity of 79%, PPV of 73%, and NPV of 83% (Table 4). Using ≤21 as the cut point increased κfrom 0.36 to 0.57.

**Table 3:** Screening accuracy of the ACT for not well controlled asthma in African-American teens

ACT score	κ	Sensitivity	Specificity	PPV	NPV	AUC
≤14	0.128	0.156	1.000	1.000	0.438	0.578
≤15	0.182	0.219	1.000	1.000	0.457	0.609
≤16	0.237	0.281	1.000	1.000	0.477	0.641
≤17	0.254	0.344	0.952	0.917	0.488	0.648
≤18	0.303	0.438	0.905	0.875	0.514	0.671
≤19	0.425	0.562	0.905	0.900	0.576	0.734
≤20	0.482	0.656	0.857	0.875	0.621	0.757
≤21	0.527	0.812	0.714	0.812	0.714	0.763
≤22	0.494	0.938	0.524	0.750	0.846	0.731
≤23	0.338	0.969	0.333	0.689	0.875	0.651
≤24	0.221	1.000	0.190	0.653	1.000	0.595

**Table 4:** Screening accuracy of the ACT for not well controlled asthma in non-African-American teens

ACT score	κ	Sensitivity	Specificity	PPV	NPV	AUC
≤14	0.161	0.143	1.000	1.000	0.613	0.571
≤15	0.315	0.286	1.000	1.000	0.655	0.643
≤16	0.390	0.357	1.000	1.000	0.679	0.679
≤17	0.329	0.357	0.947	0.833	0.667	0.652
≤18	0.402	0.429	0.947	0.857	0.692	0.688
≤19	0.355	0.500	0.842	0.700	0.696	0.671
≤20	0.494	0.643	0.842	0.750	0.762	0.742
≤21	0.570	0.786	0.789	0.733	0.833	0.788
≤22	0.530	0.929	0.632	0.650	0.923	0.780
≤23	0.371	0.929	0.474	0.565	0.900	0.701
≤24	0.281	1.000	0.316	0.519	1.000	0.658

Additionally, we conducted an analysis of baseline visit responses to each of the five ACT questions to determine if particular questions were more or less predictive of not well controlled asthma (see Additional file 1: Tables S3 and S4 in the Online Repository). Within the AA group, we found that question 1 (“*In the past 4 weeks, how much of the time did your asthma keep you from getting as much done at work, school or at home?*”) and question 3 (“*During the past 4 weeks, how often did your asthma symptoms (wheezing, coughing, shortness of breath, chest tightness or pain) wake you up at night or earlier than usual in the morning?*”) had the best screening properties for not well controlled asthma. We then examined the screening properties of every possible pair of ACT questions and again found that questions 1 and 3 produced the best results. At the baseline visit, a score of ≤9 for this “sub-test” amongst AA teens was 91% sensitive and 86% specific for not well controlled asthma, with an area under the ROC curve of 0.88. These findings were not replicated at the follow up visit for AA teens nor were similar results found at either visit for nAA teens.

### Responsiveness to change

To determine the responsiveness of the ACT questionnaire to changes in asthma control status over time, we calculated the correlation between change in ACT score and change in physician assessment of control between baseline and follow up study visits. Few participants experienced a change in asthma control status during the study period. A small but statistically significant correlation was seen between change in ACT score and change in asthma control in AA teens (*r* = 0.29, *p* = 0.04) but not in nAA teens (*r* = 0.32, *p* = 0.08). Within the nAA group only, we identified a significant difference in mean change in ACT score between participants whose asthma improved, worsened, or stayed the same (F = 3.2, *p* = 0.05) (Table 5). Change in ACT score was significantly correlated with change in FEV_1_ (*r* = 0.53, *p* = 0.002) and FEV_1_/FVC (*r* = 0.55, *p* = 0.001*)* in nAA teens but not in AA teens (ΔFEV_1_, *r* = 0.11, *p* = 0.46 and ΔFEV_1_/FVC, r = 0.2, *p* = 0.15) (see Additional file 1: Table S5 in the Online Repository).

**Table 5:** Responsiveness of the ACT to change in physician-assessed asthma control

African-American				Non-African-American			
Physician Assessment	Mean (SD) change in ACT score	F	*p*	Physician Assessment	Mean (SD) change in ACT score	F	*p*
Worsened control (*n* = 4)	−1.00 (3.56)	2.19	0.12	Worsened control (n = 5)	0.4 (2.07)	3.23	0.05
Unchanged (*n* = 37)	0.49 (3.66)			Unchanged (*n* = 19)	−0.32 (2.75)		
Improved control (*n* = 10)	2.80 (3.33)			Improved control (*n* = 8)	3.38 (5.26)		

## Discussion

Standardized patient-reported questionnaires like the ACT allow providers to quickly assess asthma control in busy clinical practices. While previous studies have provided evidence in support of the validity and reliability of the ACT for detecting suboptimal asthma control [8, 10, 25], information on how the test performs in adolescents and in ethnically diverse populations is lacking given concerns that the questionnaire may not be sensitive to how teenagers and minorities perceive asthma-related impairment. Because minorities and teens/young adults are already at higher risk of poor asthma outcomes, it is imperative to understand the value of the tools being used to drive management decisions in these groups, to avoid overestimating level of control and under-treating asthma.

Our results suggest that the currently accepted ACT cut point score of ≤19 showed reduced accuracy compared to previous studies [8, 10, 25] in identifying asthma that is not well controlled in both AA and nAA adolescents. In both groups, use of a higher cut point score of ≤21 achieved the greatest area under the ROC curve and the best balance of sensitivity and specificity for identification of not well controlled asthma. That these results were seen in both AA and nAA groups suggests that a higher ACT cut point score may be necessary in adolescents. It is well established that children and adolescents are less accurate in describing their perceived asthma control [26–32], impacted by age and developmental level. They may “normalize” their asthma symptoms and therefore not recognize them as being problematic, or they may minimize symptoms to avoid being categorized as different from their peers [33, 34]. Given these well documented differences in symptom perception and reporting, it seems logical to adjust the expectations of self-administered questionnaires that are based primarily on patient-reported data points. It is also important to emphasize that this is not a problem isolated to teenagers. In fact, using the childhood ACT (cACT) for children under 12 years, our group has published evidence that caregiver perception of asthma control may be even more discrepent from physician assessment than teen perception [9].

Analysis of individual ACT questions demonstrates that certain elements of the assessment may be more predictive of uncontrolled asthma than others in AA teens, particularly activity limitation and nighttime symptoms. This subtest requires further study to determine its potential utility as an adjunct measure of asthma control but emphasizes the importance of considering cultural context in the design of standardized questionnaires. For example, African-Americans with asthma may report less nighttime awakening and dyspnea, two symptoms that account for 20% of the ACT score [15, 26, 35]. This leads to under-reporting of symptoms, which reflects a false level of asthma control when queried by the ACT.

Our findings demonstrate acceptable levels of internal consistency reliability for both the AA and nAA teens at the group level; however, the reliability is below what is the recommended threshold for individual level assessment. This finding recognizes that there is a higher level of measurement error associated with the estimate that may caution providers to not solely rely on the ACT for determining asthma control. Further, the test-retest reliability of the ACT within the AA teen group was lower than the nAA teens. There should be some caution in overinterpreting the test-retest reliability as there was a 6–8 week time lapse between the baseline and follow up assessments; thus, patients’ stable asthma control status could fluctuate and participants’ memory of their control at baseline may be less clear.

Few participants experienced a change in level of asthma control, which was not surprising given the lack of an intervention and the brief 6–8 week study period. The responsiveness of the ACT to changes in asthma control was marginal in both AA and nAA adolescents. We found no significant correlation between change in ACT scores and change in asthma control status among nAA teens, with a small yet statistically significant correlation in AA teens. However, we found no difference in mean change in ACT scores between groups whose asthma worsened, improved, or remained stable in the AA group, suggesting that ACT scores do not significantly increase or decrease with improved or worsened asthma control, respectively. Interestingly, change in ACT score was strongly correlated with change in FEV_1_ and FEV_1_/FVC in nAA teens but very poorly correlated in AA teens, despite similar median % predicted FEV_1_ between the two groups.

The ACT did not discriminate well between participants with higher lung function and those with lower lung function, as seen in previous studies [8, 10, 36]. However, we recognize that in children especially, spirometry alone is often a poor measure of asthma control, since children often have normal spirometry despite poorly controlled asthma. Indeed in our study, the median %predicted FEV_1_ for both AA and nAA teens was greater than 90%, despite a significant proportion of our subjects having asthma that was not well controlled.

The fair to moderate agreement (κ) between the ACT and NAEPP-based physician assessment of asthma control is consistent with prior validation studies but, in our view, emphasizes the danger of relying too heavily on the ACT score for medical decision making. We argue that while the ACT using an optimized threshold score may be a useful screening tool, it should not replace a comprehensive physician assessment of control in the clinical setting. However, the context in which the questionnaire is being used may impact its utility and the choice of cut point score. For example, when used in the clinical setting, a more sensitive cut point should be chosen to minimize false negatives (persons with asthma that is not well controlled whose ACT score indicates controlled disease). Conversely in the research setting if the goal is to recruit poorly controlled asthmatics, a cut point that maximizes specificity, reducing the rate of false positives, is desired to avoid recruiting well controlled asthmatics to the study.

Accurate assessment of asthma control requires evaluation of both of the essential elements that comprise control: impairment and risk of future exacerbations. Pediatric asthma is often associated with low levels of day-to-day impairment but excessive risk in the form of frequent exacerbations, often occurring with viral respiratory infections. The ACT primarily measures impairment and does not take into account elements that increase risk (such as history of exacerbations in the prior year, unscheduled healthcare visits or hospitalizations for asthma), which may limit its utility in pediatric patients. The ACT also provides no mechanism to estimate asthma severity. The Composite Asthma Severity Index (CASI) takes into account impairment, exacerbations, controller medication requirement and lung function to provide a more comprehensive evaluation of asthma and to measure response to therapies [37]. A similar multipronged evaluation of asthma may be a better guide for stepping up or down treatment in clinical practice or for measuring asthma treatment responses in clinical research.

Our study has several limitations. The relatively small sample size increased the variance seen and did not allow us to explore sub-group differences by age (e.g., 12–15 years vs 16–18 years) to examine whether younger children had more challenges than older children in understanding the questionnaire or relating their asthma control experiences to answer the questions. All participants and their caregivers were required to speak English to participate in the study, which hindered recruitment of Latino children, another group at high risk of asthma-related morbidity. Our physician assessment of asthma control was binary (well controlled vs not well controlled) in contrast to other studies that incorporated terms such as “partly controlled”, “somewhat controlled”, or “not at all controlled,” which may have limited our ability to assess the discriminant validity of the test. Few participants had a change in their asthma control status between the two study visits, which likely limits our ability to make inferences about responsiveness of the ACT to change. While the AA and nAA teen groups were well matched in terms of age, sex, atopic status, BMI, and FEV_1_, the AA group contained a larger proportion of participants on step 5 therapy or above compared to the nAA (49% vs 24%), suggesting increased asthma severity within the AA group. To address the possibility that our study populations may have contained a higher number of “poor perceivers” of asthma symptoms relative to the general adolescent population, we recruited from both general pediatrics clinics and subspecialty clinics in an effort to achieve more generalizable results. However, replication of this study in a larger group with a wider range of disease severity is needed before these findings can be applied to asthmatic teenagers in general.

## Conclusions

We found some evidence for the validity of the ACT but not ideal levels of reliability for individual level assessment along with reduced sensitivity for detection of not well controlled asthma in adolescents with persistent asthma, independent of race. A higher threshold value to define asthma control improved the predictive properties of the ACT and requires further investigation. Additionally, AA teens’ scores were less responsive to change than nAA teens, suggesting that use of the ACT to detect changes in clinical status over time may be impaired in this population. While the ACT should be used to support the assessment of asthma control, it should not be relied upon as the sole indicator of control and should not replace the NAEPP-guidelines based physician assessment.

## Additional file

Additional file 1: **Table S1.** Standardized asthma history obtained by study physicians at both study visits. **Table S2.** Correlation between baseline ACT score and spirometry measurements (as Pearson correlation coefficients). **Table S3.** Screening accuracy of individual ACT questions for detection of not well controlled asthma in African-American adolescents. **Table S4.** Screening accuracy of individual ACT questions for detection of not well controlled asthma in non-African American adolescents. **Table S5.** Correlations between change in ACT score and change in spirometry measurements between study visits (as Pearson correlation coefficients). (DOCX 31 kb)

## Abbreviations

AA: African-American
ACT: Asthma Control Test
CASI: Curve; Composite Asthma Severity Index
FEF_25–75_: Forced expiratory flow at 25–75% of pulmonary volume
FEV_1_: Forced expiratory volume in 1 s
FVC: Forced vital capacity
ICC: Intraclass correlation coefficient
nAA: Non-African-American
NAEPP: National Asthma Education and Prevention Program
PEFR: Peak expiratory flow rate
ROC: Receiver operating characteristic
