# Supplementary material for: Age and African-American race impact the validity and reliability of the asthma control test in persistent asthmatics
Source: Respir Res. 2018 Aug 15;19:152. doi: 10.1186/s12931-018-0858-0 (PMC6094875; doi:10.1186/s12931-018-0858-0)
Supplement: Supplementary file 1 — Table S1. Standardized asthma history obtained by study physicians at both study visits. Table S2. Correlation between baseline ACT score and spirometry measurements (as Pearson correlation coefficients). Table S3. Screening accuracy of individual ACT questions for detection of not well controlled asthma in African-American adolescents. Table S4. Screening accuracy of individual ACT questions for detection of not well controlled asthma in non-African American adolescents. Table S5. Correlations between change in ACT score and change in spirometry measurements between study visits (as Pearson correlation coefficients). (DOCX 31 kb) [file 12931_2018_858_MOESM1_ESM.docx]

**Additional File 1**

| **Table S1.** Standardized asthma history obtained by study physicians at both study visits. | | |
| --- | --- | --- |
| **Asthma History Review – by MD** | **YES** | **NO** |
| 1. More than 2 days of symptoms per week |  |  |
| 2. 1 or more nighttime awakenings per week |  |  |
| 3. Activity limitation |  |  |
| 4. More than 2 days per week requiring rescue medication for symptoms |  |  |
| 5. Required oral steroid more than once in past year |  |  |
| 6. New asthma medications prescribed |  |  |
| 7. Hospitalized for asthma |  |  |
| 8. Seen in ED, urgent care, or by PCP for asthma |  |  |
| 9. Missed any school due to asthma |  |  |

| **Table S2.** Correlation between baseline ACT score and spirometry measurements (as Pearson correlation coefficients). | | |
| --- | --- | --- |
| **Spirometry** | **AA** | **nAA** |
| FEV_1_ | -0.04 | 0.18 |
| FEV1/FVC | 0.07 | 0.29 |
| FEF_25-75_ | 0.03 | 0.28 |
| *ACT*, Asthma Control Test; *AA*, African-American; *FEF_25-75_*, Forced Expiratory Flow at 25-75% of Pulmonary Volume; *FEV_1_*_,_ Forced Expiratory Volume in 1 Second; *FVC*, Forced Vital Capacity; *nAA*, non-African-American | | |

| **Table S3.** Screening accuracy of individual ACT questions for detection of not well controlled asthma in African-American adolescents. | | | | | | |
| --- | --- | --- | --- | --- | --- | --- |
| **ACT Score** | **κ** | **Sensitivity** | **Specificity** | **PPV** | **NPV** | **AUC** |
| *Q1: In the past 4 weeks, how much of the time did your asthma keep you from getting as much done at work, school or at home?*  *1=All of the time; 2=Most of the Time; 3=Some of the time; 4=A little of the time; 5=None of the time* | | | | | | |
| 2 | 0.03 | 0.03 | 1.00 | 1.00 | 0.40 | 0.52 |
| 3 | 0.24 | 0.28 | 1.00 | 1.00 | 0.48 | 0.64 |
| 4 | 0.59 | 0.72 | 0.91 | 0.92 | 0.68 | 0.81 |
| *Q2: During the past 4 weeks, how often have you had shortness of breath?*  *1=More than once a day; 2=Once a day; 3=3 to 6 times a week; 4=Once or twice a week; 5=Not at all* | | | | | | |
| 2 | 0.18 | 0.22 | 1.00 | 1.00 | 0.46 | 0.61 |
| 3 | 0.17 | 0.25 | 0.95 | 0.89 | 0.46 | 0.6 |
| 4 | 0.15 | 0.72 | 0.43 | 0.66 | 0.5 | 0.57 |
| *Q3: During the past 4 weeks, how often did your asthma symptoms (wheezing, coughing, shortness of breath, chest tightness or pain) wake you up at night or earlier than usual in the morning?*  *1=4 or more nights a week; 2=2-3 nights a week; 3=Once a week; 4=Once or twice; 5=Not at all* | | | | | | |
| 2 | 0.1 | 0.13 | 1.00 | 1.00 | 0.43 | 0.56 |
| 3 | 0.27 | 0.31 | 1.00 | 1.00 | 0.49 | 0.66 |
| 4 | 0.6 | 0.69 | 0.95 | 0.96 | 0.67 | 0.82 |
| *Q4: During the past 4 weeks, how often have you used your rescue inhaler or nebulizer medication (such as albuterol)?*  *1=3 or more times per day; 2=1 or 2 times per day; 3=2 or 3 times per week; 4=Once a week or less; 5=Not at all* | | | | | | |
| 2 | 0.12 | 0.28 | 0.86 | 0.75 | 0.44 | 0.57 |
| 3 | 0.2 | 0.59 | 0.62 | 0.7 | 0.5 | 0.61 |
| 4 | 0.3 | 0.81 | 0.48 | 0.7 | 0.63 | 0.64 |
| *Q5: How would you rate your asthma control during the past 4 weeks*  *1=Not controlled at all; 2=Poorly controlled; 3=Somewhat controlled; 4=Well controlled; 5=Completely controlled* | | | | | | |
| 2 | -0.04 | 0.00 | 0.95 | 0.00 | 0.39 | 0.48 |
| 3 | 0.33 | 0.47 | 0.91 | 0.88 | 0.53 | 0.69 |
| 4 | 0.34 | 0.97 | 0.33 | 0.69 | 0.88 | 0.65 |
| *Performance of Combined Score on “Sub-Test” of Q1 and Q3* | | | | | | |
| Combined score | κ | Sensitivity | Specificity | PPV | NPV | AUC |
| 6 | 0.16 | 0.19 | 1.00 | 1.00 | 0.45 | 0.59 |
| 7 | 0.29 | 0.34 | 1.00 | 1.00 | 0.5 | 0.67 |
| 8 | 0.60 | 0.66 | 1.00 | 1.00 | 0.66 | 0.83 |
| 9 | 0.76 | 0.91 | 0.86 | 0.91 | 0.86 | 0.88 |
| *ACT*, Asthma Control Test™; *AUC*, Area Under the Curve; *κ*, Cohen’s kappa statistic; *NPV*, Negative Predictive Value; *PPV*, Positive Predictive Value  Asthma Control Test™ ©2002 by Quality Metric Incorporated. | | | | | | |

| **Table S4.** Screening accuracy of individual ACT questions for detection of not well controlled asthma in non-African American adolescents. | | | | | | |
| --- | --- | --- | --- | --- | --- | --- |
| **ACT Score** | **κ** | **Sensitivity** | **Specificity** | **PPV** | **NPV** | **AUC** |
| *Q1: In the past 4 weeks, how much of the time did your asthma keep you from getting as much done at work, school or at home?*  *1=All of the time; 2=Most of the Time; 3=Some of the time; 4=A little of the time; 5=None of the time* | | | | | | |
| 2 | 0.08 | 0.07 | 1.00 | 1.00 | 0.59 | 0.54 |
| 3 | 0.24 | 0.21 | 1.00 | 1.00 | 0.63 | 0.61 |
| 4 | 0.37 | 0.57 | 0.79 | 0.67 | 0.71 | 0.68 |
| *Q2: During the past 4 weeks, how often have you had shortness of breath?*  *1=More than once a day; 2=Once a day; 3=3 to 6 times a week; 4=Once or twice a week; 5=Not at all* | | | | | | |
| 2 | 0.33 | 0.36 | 0.95 | 0.83 | 0.67 | 0.65 |
| 3 | 0.33 | 0.36 | 0.95 | 0.83 | 0.67 | 0.65 |
| 4 | 0.34 | 0.71 | 0.63 | 0.59 | 0.75 | 0.67 |
| *Q3: During the past 4 weeks, how often did your asthma symptoms (wheezing, coughing, shortness of breath, chest tightness or pain) wake you up at night or earlier than usual in the morning?*  *1=4 or more nights a week; 2=2-3 nights a week; 3=Once a week; 4=Once or twice; 5=Not at all* | | | | | | |
| 2 | 0.1 | 0.14 | 0.95 | 0.67 | 0.6 | 0.55 |
| 3 | 0.1 | 0.14 | 0.95 | 0.67 | 0.6 | 0.55 |
| 4 | 0.1 | 0.36 | 0.74 | 0.5 | 0.61 | 0.55 |
| *Q4: During the past 4 weeks, how often have you used your rescue inhaler or nebulizer medication (such as albuterol)?*  *1=3 or more times per day; 2=1 or 2 times per day; 3=2 or 3 times per week; 4=Once a week or less; 5=Not at all* | | | | | | |
| 2 | 0.33 | 0.36 | 0.95 | 0.83 | 0.67 | 0.65 |
| 3 | 0.62 | 0.71 | 0.9 | 0.83 | 0.81 | 0.81 |
| 4 | 0.53 | 0.93 | 0.63 | 0.65 | 0.92 | 0.78 |
| *Q5: How would you rate your asthma control during the past 4 weeks*  *1=Not controlled at all; 2=Poorly controlled; 3=Somewhat controlled; 4=Well controlled; 5=Completely controlled* | | | | | | |
| 2 | 0.24 | 0.21 | 1.00 | 1.00 | 0.63 | 0.61 |
| 3 | 0.4 | 0.43 | 0.95 | 0.86 | 0.69 | 0.69 |
| 4 | 0.26 | 0.86 | 0.42 | 0.52 | 0.8 | 0.64 |
| *ACT*, Asthma Control Test™; *AUC*, Area Under the Curve; *κ*, Cohen’s kappa statistic; *NPV*, Negative Predictive Value; *PPV*, Positive Predictive Value  Asthma Control Test™ ©2002 by Quality Metric Incorporated. | | | | | | |

| **Table S5.** Correlations between change in ACT score and change in spirometry measurements between study visits (as Pearson correlation coefficients). | | | | | |
| --- | --- | --- | --- | --- | --- |
| **AA** | | | **nAA** | | |
| **Spirometry Measurements** | **r** | ***p*** | **Spirometry Measurements** | **r** | ***p*** |
| ΔFVC | 0.02 | 0.90 | ΔFVC | 0.32 | 0.08 |
| ΔFEV_1_ | 0.11 | 0.46 | ΔFEV_1_ | 0.53 | 0.002 |
| ΔFEV1/FVC | 0.20 | 0.15 | ΔFEV1/FVC | 0.55 | 0.001 |
| ΔFEF_25-75_ | 0.11 | 0.44 | ΔFEF_25-75_ | 0.6 | <0.001 |
| ΔPEFR | 0.23 | 0.12 | ΔPEFR | 0.5 | 0.004 |
| *ACT*, Asthma Control Test; *AA*, African-American; *FEF_25-75_*, Forced Expiratory Flow at 25-75% of Pulmonary Volume; *FEV_1_*_,_ Forced Expiratory Volume in 1 Second; *FVC*, Forced Vital Capacity; *nAA*, non-African-American | | | | | |
